# Tailoring human joint-on-a-chip: from biological principles, materials, to disease modeling

**DOI:** 10.1016/j.mtbio.2025.102549

**Published:** 2025-11-13

**Authors:** Xuejiao Wang, Louis Jun Ye Ong, Marcel Karperien, Chunyi Wen

**Affiliations:** a Department of Biomedical Engineering, Research Institue of Smart Ageing, The Hong Kong Polytechnic University, Hung Hom, Hong Kong; b School of Mechanical, Medical and Process Engineering, Queensland University of Technology, Brisbane, Australia; c QUT Centre for Biomedical Technologies, Queensland University of Technology, Australia; d Max Planck Queensland Centre (MPQC) for the Materials Science of Extracellular Matrices, Queensland University of Technology, Brisbane, Australia; e Temasek Polytechnic, School of Applied Sciences, Singapore; f Department of Developmental BioEngineering, TechMed Institute, University of Twente, Enschede, 7522, NB, the Netherlands

**Keywords:** Joint-on-a-chip, Microfluidics, Joint disorders, Disease modeling, Cell co-culture

## Abstract

Joint-on-a-chip (JoC) is a miniaturized *in vitro* model based on microfluidics and tissue engineering, aiming to provide revelational platform to study joint physiology and pathology for personalized medicine. Engineering biomimetic JoC necessitates recreating the physiological architecture and biomechanical environment, while incorporating systemic risk factors involved in pathogenesis. In this review, we aim to provide an overview manual to bridge the gap between engineers, biologists, and clinicians. We start with joint biology to the basic characteristics of JoC and summarize the important designing factors. We categorize materials used in chip fabrication and highlight how recent advances in material science, integrated with established microfabrication techniques have enabled the development of advanced chips. Existing joint-on-a-chip platforms enable targeted investigation and high-fidelity modeling of disease-specific analysis through deliberately simplified, reductionist tissue interaction systems. We anticipate that the future development of joint-on-a-chip models will hinge on addressing cell co-culture, automated control, and real-time monitoring. Successfully tackling these challenges will greatly accelerate the application of JoC in foundational research, drug discovery, and personalized medicine.

## Graphical abstract

This review begins with joint biology-guided on chip joint models, critically examines the materials for chip engineering, and highlights how design considerations should align with the intended clinical purpose and biological applications.

**Figure:**
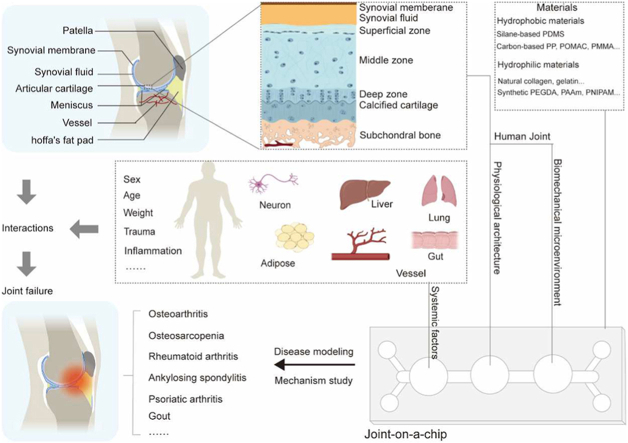

## 1 Introduction

Joint disorders such as osteoarthritis (OA) and rheumatoid arthritis (RA) are among the chronic diseases with the highest disability rates in the world [[1], [2], [3], [4]]. Their pathological mechanisms involve complex multidimensional interactive networks, including signaling pathway dysregulation [5], microenvironment dynamic imbalance [6,7] and cross-border regulation of the immune system [8,9]. The interweaving of these physiological and pathological processes makes the study of disease mechanisms, and the development of new therapies face challenges. Up to today, the development of disease-modifying therapeutics remains limited: only a few options exist for RA, and none for OA. Current clinical interventions are largely palliative, offering symptomatic relief rather than altering disease progression. A model that precisely mimic human pathology and predicts *in vivo* responses plays an important role in efficient therapeutic development. However, traditional 2D models fail to replicate the 3D structure of natural microenvironment of joint and reproduce cell-cell/matrix interactions [10,11]. Animal models enable a more physiological environment, yet their interspecies differences with humans in genetic background, immune response and microbial interaction have severely limited the translation of research results into the clinic [[12], [13], [14]].

Organ-on-a-chip (OoC) is a revolutionary microphysiological system that reconstructs the spatiotemporal dynamic characteristics of human tissues *in vitro* [[15], [16], [17], [18]]. Chips integrated with patient-derived cells can be directly adopted as individualized pathological models [[19], [20], [21]]. Following the FDA's landmark promoting *in vitro* models for drug assessment, OoC has undergone remarkable expansion and advancement [22]. Balancing the engineering controllability of the "simplified model" and the pathological authenticity of the "complex system" for disease modeling is a key breakthrough in accelerating its transition from concept verification to clinical transformation [23]. In the field of skeletal joints, many Joint-on-a-Chip (JoC) models have been developed and summarized [24]. These chips simulate the physiological and dynamic environment of different tissues of joint to study local risk factors such as mechanical compression, stress, and inflammation. In addition to local effects, joint disorders are also subject to the interplay between local and systemic risk factors, such as endothelial dysfunction [25]. Thanks to the rapid development of manufacturing and material sciences, more tissues and bioenvironmental elements can be recruited on a chip, promising in *in vitro* JoC-based disease model for mechanism study and drug screening [26].

In this review, we categorized and detailed the materials used in chip fabrication based on their hydrophilic and hydrophobic characteristics, and elucidated how materials can be leveraged, and even drive innovations in current manufacturing technologies to enable competitive JoC scaffold fabrication. In addition, we start from joint biology, examining the enabling technologies for chip engineering, highlighting how design considerations should align with the intended clinical purpose and biological applications. We anticipate that the future development of joint-on-a-chip technologies will address several critical challenges: achieving robust and reproducible cell differentiation, implementing automated systems and sensors for high-throughput processing. By advocating for a bespoke design strategy, this review provides a framework to guide researchers in developing physiologically relevant and translationally viable JoC platforms.

## 2 Joint biology

**Fig. 1:**
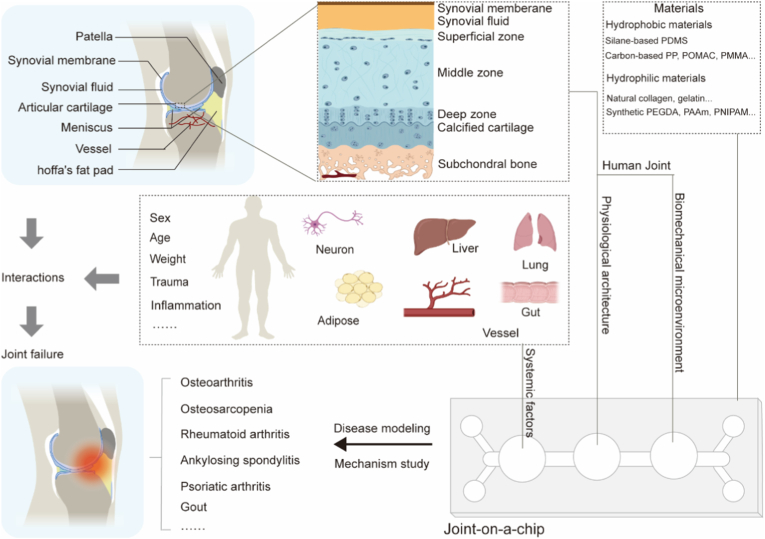
Tailoring Joint disorders-oriented Joint-on-a-chip. The physiological architecture and biomechanical environment of human joint, together with multi causative factors, orient joint-on-a-chip tailoring for joint disease modeling and mechanism investigation.

From basic research on bone biology to the construction of JoC, the essence is to transform theoretical understanding of complex physiological systems into controllable engineered entities. As is well known, the joint includes multiple tissues such as bone, vascular, articular cartilage, synovial fluid, synovial membrane, patella, and so on (Fig. 1A) [27]. The types of cells, metabolic components, and extracellular matrix vary across different tissues. Cartilage primarily consists of chondrocytes and an extracellular matrix composed of collagen fibers and proteoglycans. It functions to distribute joint stress and reduce bone wear. A structural heterogeneity exists from cartilage to bone, subdivided into the superficial zone, middle zone, deep zone, calcified cartilage, and subchondral bone. While the Tidemark separates the radial layer of subchondral bone from calcified cartilage [28]. Bone contains osteoblasts, osteoclasts, osteocytes, bone marrow, hydroxyapatite, collagen fibers, etc., and is a highly vascularized tissue [29]. In contrast, cartilage exists in an avascular environment, with a lower oxygen level <9 % physiologically [[30], [31], [32]]. Nutrient supply relies on synovial fluid and diffusion through porous calcified cartilage from the subchondral bone region. However, under pathological conditions such as inflammation or mechanical injury, blood vessels may breach the Tidemark and invade cartilage from the subchondral bone. Synovial fluid is confined within a narrow space in the joint, which is secreted by the synovium and contains hyaluronic acid, lubricin, proteoglycans, glycosaminoglycans, dissolved gases, matrix metalloproteinases (MMPs) [33]. It plays a crucial role in mechanical movement, with studies showing it reduces the friction coefficient of cartilage to as low as 10^−3^ [34,35].

Based on their etiology and pathogenesis, joint diseases can be mainly divided into the following categories: degenerative osteoarthritis, inflammatory arthritis (such as rheumatoid arthritis, gout, and psoriatic arthritis), infectious arthritis, and periarticular diseases (such as bursitis and tendinitis). The progression of joint diseases is not only caused by one risk factor but involves complex interactions among multiple tissues at the physiological and pathological levels. Therefore, when constructing joint-related *in vitro* disease models, in addition to simulating the multi-tissue joint unit and its biomechanical environment, it is often necessary to include other systemic risk factors which are originated from aging and other tissue/organ dysfunctions (such as lung, gut, vessel, and so on) to more comprehensively reproduce the microenvironment in which the disease occurs and develops.

## 3 Chip scaffold fabrication

Over the past decade, polydimethylsiloxane (PDMS) has remained the predominant material for Joint-on-a-Chip (JoC) platforms, despite the availability of diverse hydrogels designed to mimic the extracellular matrix (ECM). This section broadens the discussion on JoC scaffold engineering, systematically reviewing a wider range of material systems and engineering advances to inspire future iterations of JoC manufacturing. Concurrently, we summarize the key properties of relevant materials and, by linking them to specific processing technologies, demonstrate how rational material selection enables advanced chip fabrication.

### 3.1 Engineering strategies

**Fig. 2:**
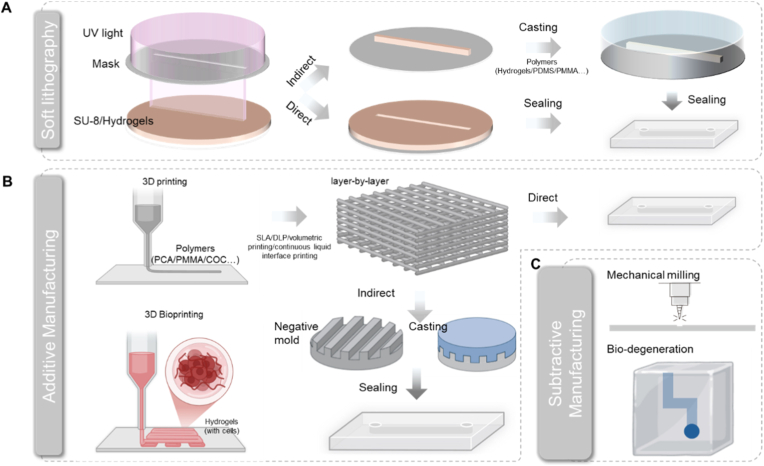
Material candidates in chip engineering. (A), Soft lithography: direct packaging of SU-8 [49] or polymer casting [50]. (B), 3D printing of different polymers and cell-hydrogel ink for chip fabrication [[51], [52], [53], [54]]. (C), Subtractive manufacturing can be realized by mechanical milling [45] or bio-degeneration [55].

Fig. 2 shows the representative manufacturing technologies including milling, soft lithography, and 3D printing with possible material candidates for preparing the scaffold of a chip. In general, chip engineering can be simply classified into two workflows, with one casting on a master mold with manufactured structures and the other directly engineering materials to chips. Following the chip is clamped for perfusion through, for example, chemical bonding, mechanical pressing, or sealing with leak-proof rubber tubes.

Soft lithography is the gold standard for constructing high-resolution structures of microfluidic chips [36,37]. It begins with the two-dimensional pattern design of the microfluidic network by computer-aided design (CAD). This design is then transferred onto a silicon wafer via ultraviolet lithography, which patterns a photoresist layer (e.g., SU-8) to create a master mold (Fig. 2A). Subsequently, polymers or hydrogels are cast against this master mold to replicate the microchannel structure. The resolution achievable through this technique typically ranges from 5 to 200 μm [38].

Additive Manufacturing (AM) is a process of fabricating three-dimensional (3D) objects through the controlled layer-by-layer deposition of materials based on a digital model (Fig. 2B) [39]. Typical materials employed include thermoplastic polymers and hydrogels that are curable via photothermal processes [40,41]. Photocuring 3D printing technology can achieve layer-by-layer stacking based on the light-induced cross-linking mechanism upon digital patterns, enabling entity construction in a short time [42]. Customized 3D entities can directly customize the negative mold of the microfluidics, which greatly reduces the dependence on clean laboratories and skilled operators compared to soft lithography.

The defining characteristic of subtractive manufacturing lies in its direct physical material removal to achieve its final form, where its flexibility, high precision, and broad material compatibility secure its critical role in precision engineering (Fig. 2C) [43]. As a quintessential subtractive process, micro-milling has gained prominence in recent years as a favored strategy for fabricating complex microstructures in organ-on-a-chip devices [44]. Governed by high-precision computer numerical control (CNC), micro-milling employs cutting tools operating within Cartesian coordinate systems to create high-resolution 3D structures—such as pillars and cavities—with varying heights and dimensions in a single continuous process [45]. Leveraging diverse tool geometries (e.g., ball-end mills for circular profiles, engraving tools for square or triangular channels), micro-milling enables the fabrication of multi-scale fluidic architectures [46]. In attempt to achieve bidirectional repeatability at sub-micron levels, multi-axis micro-milling system facilitated 3D microfluidic channel prototyping on non-planar geometric surfaces [47]. A comparison made by Baranowska (Sokolowska) et al. evidenced that micro-milling outperformed 3D printing in fabricating smooth microfluidics [48].

### 3.2 Hydrophobic materials in chip engineering

The advantage of hydrophobic materials in biomedicine lies in their anti-biofouling capability. This advantage is crucial in organs-on-chips, especially when long-term cell culture is involved. Silane-based materials and carbon-based materials are two kinds of non-wettable-to-water materials that are commonly used for compartments to support cell culture. For the former, the representative PDMS has gained a lot of interest due to its high transparency and good biocompatibility [56]. For the latter, the molecular structure of pure carbon-based materials consists only of carbon and hydrogen (belongs to polyolefins), with polypropylene (PP) as a representative in chip fabrication [57], which has strong hydrophobicity. While maintaining the main chain as C-C or carbon heteroatom bond, the side chain can be modified with polar groups, such as Poly(methyl methacrylate) (PMMA) [58,59], poly(octamethylene maleate (anhydride) citrate (POMAC) [60]. These materials are wettable yet exhibit low water uptake, a surface property that promotes increased cell adhesion. Organ-on-a-chip made of these materials have stable mechanical properties and are therefore often used in subtractive manufacturing techniques. Fabrication can be achieved through two main routes. One method involves using processed materials as negative molds for PDMS casting. Alternatively, microchannels can be directly engraved into thermoplastic materials like cyclic olefin copolymer (COC) [61] and polysulfone [62]. These materials are valued for their optical transparency, and this direct engraving approach significantly reduces fabrication time.

3D printing is outstanding in chip fabrication because of the speed, flexibility, and cost benefits [63]. Direct printing of UV-curable materials (e.g., hydrophobic Nylon, organic resins at 365/405 nm) enables the fabrication of microfluidic chips themselves or the creation of negative molds. For instance, Li et al. reported a 3D printed miniJoint chip using E-shell 450 photopolymer ink to serve as microtissue modules [64]. The intrinsic properties of the materials directly influence the structural integrity, transparency, mechanical hardness, surface smoothness and overall feasibility of post-fabrication processes for the chip. For example, when printing hollow channels, the hollows are susceptible to collapse under gravity, making fabrication challenging without temporary support. This can be overcome by using acetoxy silicone with a yield strength sufficient to prevent creep within a certain angle range, so that collapse will not occur when constructing a three-dimensional hollow channel and avoid contamination of the substrate by the uncured oil film [51]. In addition, sacrificial materials are promising in making hollow structures. Recently, Sundaram et al. [52] used functional liquid metal as a sacrificial material to prepare complex microchannel models. The high surface tension of liquid metal can be greatly reduced by adjusting the alkalinity of the solution, making it easier to enter tiny channels. With a temperature-regulated solidification and liquefaction, liquid metal was successfully used as a self-sacrificial material to prepare an intricate microvascular model.

### 3.3 Hydrophilic materials in chip engineering

The human body is a predominantly aqueous environment, making the use of hydrophilic materials essential for many biomedical and lab-on-a-chip applications. Such materials help maintain native-like hydration, which is critical for supporting biological processes. Furthermore, hydrophilic surfaces promote improved cell adhesion, spreading, and proliferation, while also reducing non-specific protein adsorption and minimizing air bubble retention due to their high surface energy.

Hydrogels are particularly suitable for chip fabrication due to their intrinsically hydrophilic nature [65]. Their polymer chains contain abundant hydrophilic functional groups that enable substantial water uptake through hydrogen bonding. The resulting water content plays a key role in determining the hydrogel's swelling behavior and mechanical properties [66]. Hydrogels are broadly categorized into two types: physically crosslinked and chemically crosslinked. Physically crosslinked hydrogels are often derived from natural polymers. Protein-based examples include collagen, gelatin, fibrin, and silk fibroin, while polysaccharides such as chitosan, alginate, and hyaluronic acid are also commonly used. These materials form networks through reversible interactions such as ionic bonds, hydrogen bonding, or molecular entanglements [67]. Although these physical crosslinks are relatively weak and responsive to environmental changes, they enable reversible gelation under mild conditions. In contrast, chemically crosslinked hydrogels typically exhibit greater mechanical strength and stability due to permanent covalent bonds. For example, some natural hydrogels like fibrin can form through enzymatic crosslinking. While many natural polymers can be chemically modified to achieve covalent networks. For instance, functionalization with methacrylate groups—yielding materials such as gelatin methacryloyl (GelMA) and methacrylated hyaluronic acid (HAMA)—allows photo-initiated crosslinking under ultraviolet light. In addition to natural polymers, other synthetic polymers such as Poly(ethylene glycol) diacrylate (PEGDA), Polyacrylamide (PAAm), and Poly(N-isopropylacrylamide (PNIPAM) showed excellent biocompatibility and processability for bone tissue engineering [68].

Hydrogels can be used as cell seeding matrixes that are injected into chip compartments. Or, it can be directly used as chip materials through casting or injection (either mixed with or without cells). Qiu et al. casted chitosan-gelatin composite hydrogel on silicon wafer master fabricated by soft lithography [49]. The hydrogel-based chip forms a perfumable microvascular network with a high precision of up to 20 μm and supports long-term cell culture up to months. While using an injectable hydrogel embedded with living cells, an organ-on-chip can be directly printed with spatial cell arrangement [69]. No single hydrogel typically fulfills the diverse material requirements for injection, including rheological properties, mechanical stiffness, and cell adhesiveness. Consequently, recent research frequently utilizes hybrid hydrogel systems comprising multiple components. Wang et al. reported a tough hydrogel ink made of sodium alginate and gelatin (or GelMA) for vascular printing through microfluidic engineering [53]. The printed hollow channels can simulate the mechanical properties, perfusion, barrier properties of human blood vessels. Additionally, the inherent photocurability of many hydrogels permits their application in layer-by-layer 3D printing, a process that is typically more efficient than extrusion printing. Recently, Collins et al. proposed a printing method on a dynamic interface, which can directly print tiny organs by using soft hydrogels [54]. This method constrains the gas-liquid interface through acoustic waves and quickly creates an unsupported structure at the dynamic interface. The relatively softness of hydrogels inhibits their use in subtractive processing. Interestingly, research has revealed that they can be processed via biochemical engineering strategies, utilizing the enzymatic degradation of the gel network. A degrading agent, collagenase, was modified on magnetic nanoparticles [55]. Collagenase sculptures the methacrylate gelatin hydrogels along the moving path of the magnetic nanoparticles, creating channels in hydrogels.

In addition to being used as chip material, hydrogel can also be used as a master mold for preparing chips. For smart hydrogels, the crosslinking and degradation can be precisely regulated using external stimuli. Therefore, hydrogels can serve as reversible photoresists: like in soft lithography, a photomask can direct light-mediated curing to form customized gel patterns. Unlike conventional photoresists, these hydrogel structures are dynamically reversible and can be altered or erased on demand through appropriate stimuli. For example, alternating irradiation of visible light (VIS) and ultraviolet light (UV) reshaped an acrylamide/azobenzene-cyclodextrin (AM/AZO-CD) hydrogel mold topology for recycling [70].

### 3.4 Material candidates for JoC scaffold engineering

A variety of materials have demonstrated potential for application in various organ-on-a-chip models due to their unique performance advantages and are expected to play a more significant role in constructing the structural framework of joint-on-a-chip (JoC) (Table 1). First, as the most adopted material, PDMS has high surface energy, so that it is not easily contaminated by bacteria, viruses, etc., but at the same time, cells have low adhesion to the surface, so it needs special treatment to improve cell adhesion. PDMS features excellent elasticity which makes it a deformation unit to be integrated into chips for compression [71].

**Table 1:** Material candidates of JoC scaffold.

Materials	Properties	Advantages	Disadvantages	Promising in JoC
Polydimethylsiloxane-PDMS	Hydrophobic Hydrophilic	Low cost, Optical transparent; High Elasticity; Oxygen permeable; Biocompatible;	Absorption of small hydrophobic molecules; Non-degradable; Limited scalability;	Deformable film for compression; Patterned surfaces for cell responses control:
Thermoplastics (including PMMA, COC, PP, POMAC)		Biodegradable; Biocompatible; Easily surface modification; Smooth surface; Optical transparent;	Limited deformability; Limited permeability;	Tough bone/calcified cartilage mimicking. Standard production; Minimized molecular absorption for drug screening
Hydrogels (including alginate, GelMA, HAMA, PNIPAM, PAAm, PEGDA)		Biocompatible. Biodegradable; Enhanced permeability;	Mechanically week and unstable.	Promotes cell adherence; Tunable mechanical properties for soft tissue (vascular/cartilage) mimicking

Thermoplastic materials offer excellent biodegradability and biocompatibility, and their surface chemistry is more amenable to biofunctionalization than PDMS. Hydrophilic thermoplastic materials effectively reduce nonspecific adsorption of hydrophobic biomolecules, thereby improving detection accuracy in drug screening experiments. Furthermore, their smooth surface and high optical transparency facilitate *in situ*, real-time observation of cellular physiological states. Despite their limited inherent deformability, they possess a natural advantage in simulating hard tissues such as bone and calcified cartilage with high Young's modulus.

Hydrogel materials exhibit higher permeability, which is conducive to the diffusion and exchange of nutrients required for cell metabolism. Although its mechanical properties can be controlled within a certain range by adjusting the gel components, its overall mechanical strength is still significantly lower than that of most synthetic polymers. However, the ability of hydrogels to promote cell adhesion has been fully verified, and its soft mechanical properties are more suitable for simulating the bionic microenvironment of natural cartilage, which has been widely reported for cartilage tissue engineering [72]. In addition, models of common vascular structures in the joint area (such as intraosseous blood vessels and synovial blood vessels) are also mostly constructed by embedding endothelial cells in the hydrogel three-dimensional skeleton.

## 4 Fundamental traits of joint-on-a-chip

### 4.1 Compartments for cell co-culture

**Fig. 3:**
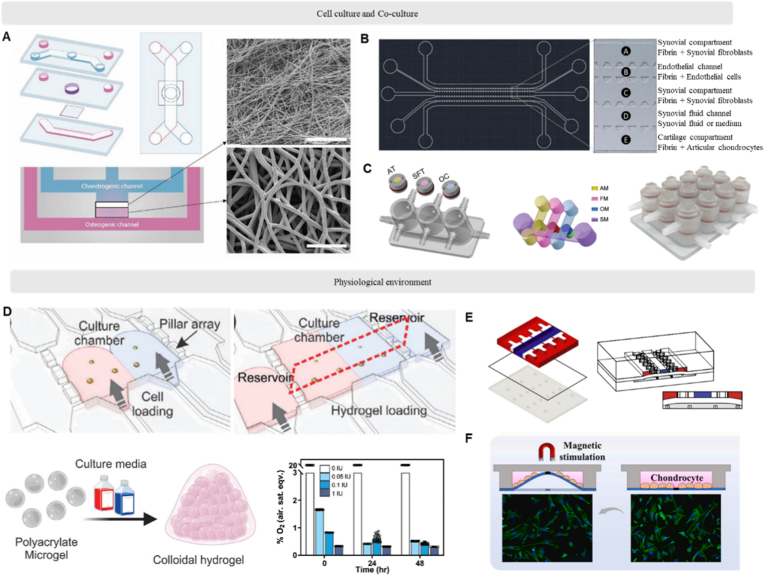
**Design factors of Joint-on-a-Chip.** (A), Porous film functions as penetration barrier between two tissue channels [75]. Copyright © 2022 The Authors. Advanced Materials Technologies published by Wiley-VCH GmbH. (B), Arrayed microstructures restrict the interaction between two tissues [81]. Copyright © 2024 IOP Publishing. (C), Perfusing channel connected multi-tissue crosstalk [64]. Copyright © 2022 The Authors. Advanced Science published by Wiley-VCH GmbH (D), Creating environmental gradients with materials on a chip [79]. Copyright © 2024 The Authors. Advanced Functional Materials published by Wiley-VCH GmbH (E), Stimuli-assisted dynamic environments [71]. Copyright © 2019, Springer Nature. (F), Smart materials for force loading [82]. Copyright © 2025 Elsevier B.V.

Different tissues in the joint possess their distinct culture media to maintain their phenotype. For instance, osteoblast differentiation relies on high calcium and phosphate concentrations, alongside osteogenic supplements like dexamethasone, β-glycerophosphate, and ascorbic acid to promote matrix mineralization [73]. Therefore, a microfluidic model with physical boundaries that isolates compartment for specific tissue culture is important. Porous membrane is one of the popular boundaries that are inserted between different compartments. The pores usually have a diameter of less than 10 μm, which is sufficient to block cell immigration while allowing for nutrients penetration. The fabrication of advanced polymeric and biological porous membranes has been achieved through diverse methods soft lithography, dry etching, high-pressure saturated steam, 3D printing, and electrospinning [74]. In the field of JoC, Meulenbelt et al. reported a microfluidic subchondral unit model that integrated a fibrous polycaprolactone matrix to set up neo-bone and neo-cartilage region (Fig. 3A) [75]. A nanofiber layer prevents cell immigration to another compartment. Alternatively, a physical boundary of arrayed micropillars can be integrated into a chip (Fig. 3B) [76]. These micropillars fix cell matrix (hydrogel) to avoid cell immigration, and their spacing enables cell crosstalk through biochemical signals' interaction. In addition, cells can be incubated independently and are connected in series. For example, Li et al. reported demonstrated a miniJoint system with a design of separate but interconnected chambers for co-culturing different tissues (Fig. 3C) [64].

### 4.2 Functional unit mimicking physiological environment

In addition to mimicking physiological structure, chip design must also account for the distinct biochemical microenvironments of joint (Fig. 3D). To achieve gradient control *in vitro*, existing organ-on-a-chip models commonly utilize asymmetrically designed perfusion channels to establish the required biochemical gradients [77,78]. Across the joint, the cartilage-to-bone axis is characterized by a gradient of increasing oxygen tension, attributed primarily to the distribution of blood vessels. For instance, Ong et al. developed a dual-perfusion chip model. By introducing an oxygen-scavenging agent, they successfully established an oxygen gradient to mimic the cartilage-to-bone interface [79].

The design philosophy of JoC differs from that of other OoCs. The core function of many OoCs is to simulate barrier function or endocrine/metabolic function. Their mechanical environments are typically fluid shear at static or cyclical tension. In contrast, JoC focuses on simulating the physiological mechanical environment of joints, which includes multimodal, cyclic mechanical loading. Enabled by the deformability of PDMS, a thin PDMS membrane was integrated into the chip. The application of pneumatic pressure induces deformation of this membrane, thereby generating compressive strain on the encapsulated hydrogel (and cells within the culture chamber). This approach has been demonstrated to achieve supraphysiological compression levels exceeding 20 % (Fig. 3E) [71]. Beyond PDMS, smart materials can be adopted in JoC to simulate compression loading. For instance, NdFeB integrated Poly-GelMA-HAMA matrix deforms under magnetic field (Fig. 3F) [80]. This model was used to study the expression of inflammation-related genes and proteins in chondrocytes upon compression.

## 5 JoC co-culture models mimic joint diseases

**Fig. 4:**
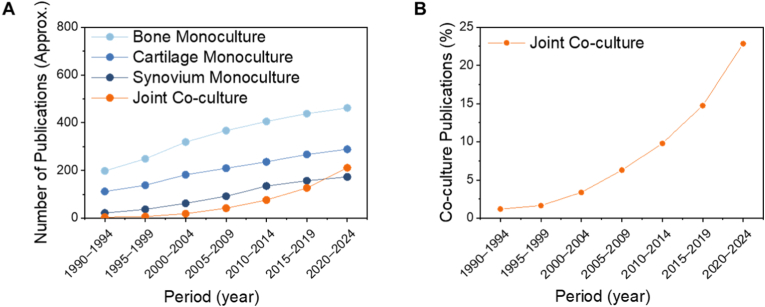
**Reported works on microfluidic models for joint tissue culture covering cartilage, bone and synovium monocultures and co-culture models.** Relevant articles were identified through a targeted search in the PubMed and Web of Science databases, covering January 1990 to March 2024. Keywords used: cartilage culture, bone culture, synovium culture, synoviocyte culture, cartilage co-culture, bone co-culture. A, number of reports identified. B, Relative abundance of reports for co-culture literatures over the total number of identified reports.

With the growing understanding that joint disorders often involve interactions between multiple tissue types, recent literature has increasingly reported co-culture systems (Fig. 4) that aim to recapitulate these complex interactions. In Table 2, we summarized the recent developed JoCs regarding tissues, cell types, materials, and applications.

**Table 2:** Joint disease-oriented on chip models within 5 years cover key aspects of joint physiology.

Joint disorders	Tissue	Cell type	Mechanical environment	Materials	Application	Reference
Osteoarthritis (OA)	Cartilage-bone	Human: Primary osteogenic cells and hPACs	No	PDMS Polycaprolactone	Interaction between cartilage and bone	[75]
	Cartilage-bone	Human: Primary chondrocytes, osteoblast	No	PDMS Polyacrylate microgel	Oxygen regulated environment reveals hypoxia influenced joint	[76]
	Cartilage-synovium	Human: Primary synovial fibroblasts; primary chondrocytes	No	PDMS Fibrin collagen gel	Synovium influence on OA pathogenesis	[114]
	Cartilage-synovial fluid-synovium	Human: MSC; Chondrocytes; synovial fibroblasts	No	PDMS Fibrin gels (synovial fibroblast) HA-PEGDA (Chondrocytes)	Patient-specific model for drug screening	[93]
	Cartilage-bone-vessel	Human: Chondrocytes; Osteoblast and osteoclast (differentiated from BMSCs); endothelial cells;	No	PDMS Fibrin gel	Vascularized osteochondral unit for disease modeling and drug screening	[115]
		Human mesenchymal stem cells (hMSCs)	No	PDMS Gelatin	Vascularized microfluidic model modeling inflammatory response and therapeutic screening	[21]
	Cartilage-synovial fluid-synovium -vessel	Human: Chondrocytes; synovial fibroblasts; endothelial cells; monocytes; synovial fluid	No	PDMS Fibrin gel	Monocyte extravasation on OA progression	[81]
	Cartilage-bone-adipose-synovial-like fibrous tissues	Human: hBMSC	No	Photopolymer ink; GelMA	Multi-tissues in joint for drug screening	[64]
	Cartilage	Human: Chondrocytes;	Yes	PDMS Agarose	Multi-directional mechanical stimulation induced OA phenotype	[116]
	Cartilage	C28/I2 cells	Yes	PDMS GelMA, HAMA, NdFeB	Osteoarthritis recruitment	[82]
	Cartilage-bone	Human: Chondrocytes; hBMSC	Yes	PDMS Poly (ethylene glycol) (PEG)	Mechanism research: release and accumulation of calcium crystals at osteochondral interface	[94]
Rheumatoid arthritis (RA)	Chondro-synovial	Human primary chondrocytes; primary FLS cells	No	PDMS	Reciprocal cross talk studies in arthritis research	[113]

### 5.1 On-chip models for osteoarthritis

Osteoarthritis (OA) is a debilitating degenerative joint disorder, which affects millions of people worldwide, including 18 % of women and 10 % of man for an age 60 or above [83]. It is clinically defined by chronic joint pain, articular cartilage degradation, synovial inflammation, and pathological bone remodeling [84]. In animal models, cyclic compression at middle and high magnitude has successfully induced OA-like phenotype, including cartilage degradation and osteophyte formation [85]. However, the etiology of OA is complex and multifactorial, which is far beyond mere mechanical stress and attrition. Among the cell populations, the homeostasis of chondrocytes plays the most pivotal role in the pathogenesis of OA. The mainstream theory underlies that the inflammatory cytokines (TNF-α, CCL2, IL-1β, etc.) and extracellular matrix modifiers (MMPs, etc.) secreted by chondrocytes are major contributors: the hyperactive inflammatory cytokines recruit immunes to erroneously attack joint tissues and cause inflammatory damage. Even worse, the inflammatory cytokines can self-amplify by activating downstream pathways, driving a vicious cycle [86]. Metalloproteinase-13 (MMP-13) is thought to be a major driver of OA by degrading the type II collagen, leading to cartilage breakdown. The breakdown products of cartilage stimulate the release of inflammatory cytokines and MMPs to enhance the vicious cycle [87].

Several joint-on-a-chip platforms have been established that utilize mechanical stress or inflammatory factors to induce disease-like phenotypes for screening applications. Concurrently, these models are evolving from simplistic cartilage-focused designs to sophisticated multi-tissue platforms that incorporate synovium, synovial fluid, cartilage, and bone to better mimic the joint's pathophysiological complexity (Table 2). Zhao et al. induced an OA phenotype with interleukin-1 beta (IL-1β) using a cartilage-on-a-chip model for OA treatment [88]. In addition, a cartilage-synovial chip facilitates cartilage degeneration response to synovitis. The chip usually coculture chondrocytes and synoviocytes on microfluidic chips, with perfusion simulating a dynamic liquid actuation [89]. Creating a simplified bone-cartilage chip benefits the investigation on cartilage degeneration mechanism through bone remodeling [[90], [91], [92]]. Recently, Ong et al. reported a bone-cartilage chip, which leverage on functionalizing shear-yielding hydrogels with sodium sulfite, an oxygen scavenger to simulate the physiological environment [76]. They found that sclerotic osteoblasts modulate chondrocyte collagen expression via MMP13 and ADAM15 secretion, an effect that is oxygen tension dependent. Patta et al. reported a microfluidic joint-on-a-chip model recruiting human primary chondrocytes in HA-PEGDA, synovial fibroblast in fibrin gel, and perfusing synovial fluid, which serves as a personalized drug screening platform [93]. Li et al. recruited more tissues including cartilage, bone, adipose, synovium on a chip. They adopted used the proinflammatory cytokine IL-1*β* to create “synovitis” [64]. Their results showed that the osteochondral unit exhibited decreased ACAN and COL2 expression, and significantly upregulated IL1B as well as MMP-3 & 13. Instead of personalized OA on-chip models and inflammatory-induced OA-like models, current chips also use compression to induce OA phenotype for drug screening. Occhetta et al. has demonstrated the hyper physiological compression inducing OA phenotype with a cartilage-on-a-chip model [71]. Recently, this model has been further updated to an osteochondral unit-on-chip platform that is capable of strain-controlled, tissue (cartilage) specific compression [94]. The release and accumulation of calcium crystals increased upon hydro-physiological loading.

Obesity plays a multifaceted role in the progression of OA. Adipose tissue dysfunction and metabolic syndrome have shown intricate influence on OA [95,96]. In a pivotal *in vivo* study, Collins et al. demonstrated that mice lacking adipose tissue due to lipodystrophy exhibited protection against OA [97], even when subjected to high-fat diets or post-traumatic joint damage. Notably, reintroducing a small fat pad into these mice reinstated their OA susceptibility, reinforcing that adipose tissue itself contributes directly to OA pathogenesis. While this study highlighted the systemic importance of adipose-derived signals, the exact molecular mediators were not directly profiled. Complementary insights in reviews have summarized how key adipokines such as leptin, resistin, and adiponectin modulate joint health through pathways including NF-κB, JAK/STAT, MAPK, and PI3K/Akt [[98], [99], [100]]. These adipokines promote inflammatory signaling and upregulate matrix-degrading enzymes (e.g., MMPs), collectively shifting chondrocytes toward a catabolic phenotype. Interestingly, adiponectin may have a dual role, with activation of the AMPK/mTOR pathway supporting autophagy and mitigating inflammation under certain conditions. Despite these mechanistic advances, the absence of co-culture platforms that integrate functional remainytes and chondrocytes within microfluidic environments remains a bottleneck. Emerging approaches in biomaterial which describe scaffold-based strategies to generate vascularized adipose tissue *in vitro*, may provide a crucial enabling technology to bridge this gap and advance cartilage–adipocyte co-culture within Organ-on-a-Chip frameworks [101].

Despite the absence of intrinsic vasculature in cartilage, clinical cohort studies reveal a strong association between vascular dysfunction and OA severity, suggesting extrinsic vascular involvement in chondral degeneration. For instance, abnormal activation of endothelin, renin-angiotensin, and Wnt-β-catenin signaling pathways in vascular can induce phenotypic changes in chondrocytes and trigger cartilage degradation [[102], [103], [104]]. Furthermore, clinical data show that hypertension increases intraosseous pressure, which leads to abnormal subchondral bone perfusion and ischemia at the tissue level, thereby impairing the integrity of the bone-cartilage functional unit [25,105]. Recently, JoC recruits co-culture of endothelial cells and chondrocytes to simulate vascularized cartilage, which serves as OA disease model for drug repurpose and development [106,107]. Yet, the field critically lacks vascular-cartilage crosstalk models capable of investigating the direct causal relationship between vascular dysfunction and cartilage degeneration. It is challenging to achieve a physiological difference [108,109]. Moreover, current vascular models, despite their proliferation, fail to faithfully reproduce the physiological dynamics of vascular regulation present in living systems.

### 5.2 On-chip models for inflammatory joint diseases

Inflammatory joint diseases are characterized by chronic joint inflammation caused by dysregulated immune system activation, which can lead to pain, swelling, fatigue, and progressive structural damage [[110], [111], [112]]. Common immune-related joint disorders include rheumatoid arthritis (RA), ankylosing spondylitis (AS), and psoriatic arthritis (PsA). During the disease process, excessive inflammatory factors, such as interleukin-6 and tumor necrosis factor, are produced, which can lead to synovial cell proliferation and ultimately cartilage destruction and bone erosion. Therefore, synovium-based joint chips are crucial for studying inflammatory joint diseases. Co-culturing chondrocytes with synovialcytes demonstrated improved chondrogenesis, suggesting potential tissue crosstalk contributing to RA disease progression. A study by Rothbauer et al. established a 3D co-culture model of chondrocytes and synoviocytes, replicating the inflammatory milieu of rheumatoid arthritis and serving as a tool for drug screening [113].

### 5.3 Pain in joint-related diseases

Pain is a hallmark symptom of joint diseases [[117], [118], [119], [120]], yet most *in vitro* joint models do not account for nociceptive signaling. The incorporation of neurons into joint-on-a-chip systems offers an opportunity to mechanistically evaluate pain responses and screen analgesic compounds in a tissue-relevant context. Recent neuron–cartilage co-culture models have begun to address this gap, with studies demonstrating that chondrocyte-derived inflammatory mediators can sensitize sensory neurons and modulate their excitability [[121], [122], [123]].

Key markers associated with nociceptive activation include calcitonin gene-related peptide (CGRP), substance P, TRPV1, and Nav1.8, all of which are commonly upregulated in dorsal root ganglia (DRG)-derived sensory neurons in response to inflammatory cytokines such as IL-1β and TNF-α secreted by stimulated chondrocytes [124]. For instance, co-culture of DRG neurons with IL-1β-stimulated chondrocytes has been shown to induce neurite sprouting and increased CGRP expression [125], suggesting a direct link between chondrocyte inflammation and neuronal sensitization. However, chip designs that support both chondrogenesis and functional nociceptor formation remain technically demanding. Innervation of neurons typically requires the fabrication of microchannels to guide and facilitate the innervation and maturation process. As such, design of microchannel arrays down to 4 μm are typically required for the innervation process [126]. Due to harvesting pre-differentiated neurons disrupts the spatiotemporal patterns of the functional neurons and the fabrication limitation to align microchannels over innervated neurons, it is always the case that incorporation of neuronal co-culture requires in-chip differentiation. Therefore, functional sensory neurons require *in situ* seeding and differentiation to support axonal outgrowth and stable neural network formation. This demands precise spatial and temporal control of neurotrophic factors such as nerve growth factor (NGF), brain-derived neurotrophic factor (BDNF), and glial cell line-derived neurotrophic factor (GDNF). These factors must be presented in a controlled gradient to direct neurite extension and preserve sensory neuron phenotypes over prolonged culture periods. Emerging technologies now offer strategies to engineer these gradients in a controllable and modular fashion. For example, Morsut's group recently developed a technique catering to pattering of synthetic notch receptors using a hydrogel system that allows spatially confined delivery of growth factors, offering a practical route for patterned stimulation [127]. Such systems could be adapted within microfluidic chips to maintain a neurotrophic niche in one compartment while permitting chondrogenic induction in another.

### 5.4 Multi-tissue/organ co-culture models for joint health

Beyond local interactions, systemic influences from distant organs also modulate joint health (Table 3). The gut-joint axis hypothesis posits a potential interaction between the gut and joint. Multiple factors associated with osteoarthritis, such as aging, gender, diet, and obesity, can contribute to gut microbial dysbiosis, which has been shown to be a potential risk factor for the development of OA and RA [128]. When the gut microbiome is dysbiotic, proinflammatory metabolites (such as lipopolysaccharide) produced by the gut microbiome can enter the systemic circulation through the increased permeability of the intestinal barrier, triggering low-grade systemic inflammation. Given that elevated lipopolysaccharide levels are closely associated with obesity and metabolic syndrome—both significant risk factors for osteoarthritis—it is reasonable to speculate that the gut microbiome contributes to the pathogenesis of osteoarthritis, at least in part, through lipopolysaccharide-mediated metabolic endotoxemia, macrophage activation, and subsequent joint tissue destruction. Existing studies have demonstrated an association between specific gut microbes and osteoarthritis progression [129] and pain. Recently, Liu et al. proposed a human microphysiological system which recruits microbe-gut and joint on a chip to study the crosstalk under OA-like conditions [130].

**Table 3:** Potential co-culture models uncovering mechanism joint physiology in relation to the body systems.

Systemic Pair	Relation to Joint Health	Reference
Adipose Tissue–Joint Axis	Adipokines like leptin and adiponectin modulate cartilage catabolism and synovial inflammation.	[128,134]
Gut–Joint Axis	Gut dysbiosis is linked to RA and OA onset through systemic inflammation and immune priming.	[128,135]
Liver–Joint Axis	Liver-derived inflammatory molecules (e.g., CRP, serum amyloid A) aggravate synovitis.	[134,135]
Bone Marrow–Joint Axis	Bone marrow niches produce immune cells contributing to chronic joint inflammation.	[136]
Circadian rhythm	In rat models of collagen-induced arthritis, the circadian rhythms of HPA axis hormones like corticosterone are disrupted, correlating with fluctuations in pro-inflammatory cytokines such as TNF-α and IL-6. This suggests that inflammation can alter circadian hormone patterns, potentially exacerbating joint disease symptoms.	[137]
Neuron–Cartilage/Neuron–Joint Tissue	Integrates sensory neurons to assess pain signaling in joint diseases.	[122]
Cartilage-Vessel	Vascular endothelial dysfunction drives OA progression	[5,25]

There is an etiopathogenic link between liver diseases and joint disorders. Patients with post-traumatic joint injury are more likely to have abnormal liver function. This abscopal effect could be due to the adoptive transfer of activated CD4^+^ T cells from the peripheral blood [131]. Liver metabolites are involved in the progression of RA. Zhao et al. demonstrated a positive correlation between the hepatotropic virus hepatitis B virus (HBV) and RA [132]. The results indicated that HBV-encoded HBx upregulates TRAFD1 to promote FMT in RA. So far, there is no reported organs-on-a-chip targeting the axis between liver-blood vessel-joint. While a liver-bone co-culture system established by Chen et al. has shown feasibility for drug toxicity screening [133]. The co-culture system could maintain a long-term stable system of liver spheroids and bone scaffolds up to 21 days.

Although systemic JoC platforms are still emerging, preliminary research highlights several key axes of interest. In Table 3, we exemplified the influence of organs from neuron, gut, liver, to circadian rhythm on the joint health, aiming to recapitulate multi-organ crosstalk affecting joint pathophysiology.

## 6 Future development of JoC

**Fig. 5:**
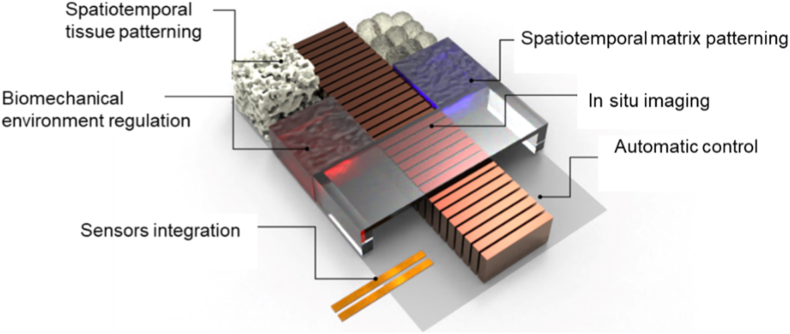
Future development of JoC requires attention on spatiotemporal tissue patterning, spatiotemporal matrix patterning, biomechanical environment regulation, *In situ* imaging, sensors integration, and automatic control.

Current joint-on-a-chip models predominantly focus on one or two tissue types with simplified microenvironmental controls. Such reductionist approaches help to isolate specific tissue interactions by minimizing confounding factors. Nevertheless, a physiologically relevant vitro joint model that integrates all major articular components is challenging, while it would significantly advance our understanding of both joint physiology and pathological mechanisms. Given the complexity of integrating all these features into a single device, most JoC platforms prioritize specific application needs, leading to diverse and modular designs. Until today, we have yet to see one JoC design that successfully demonstrated integrations of all features. While smart design strategies can partially mitigate integration challenges, physical and material constraints often limit the simultaneous implementation of all desired features. Future improvements in materials, modular interfaces, and fabrication processes will be crucial to achieving more comprehensive platforms. In this section, we discussed the remaining challenges that limit broader translational use in detail (Fig. 5).

### 6.1 Multi-tissue co-culture

The inter-tissue interactions dictate the behavior and pathophysiology of joint diseases. Therefore, it is important to capture all, if not, essential tissue types to enable accurate study and drug screening. For example, during OA, the dysregulated osteoblast contributes to the subsequent degradation of the cartilage matrix by the neighboring chondrocytes by changing their regulation of metalloprotease-mediated matrix degradation and collagen production [76,138]. In addition, replicating the intricate architecture and temporal progression of joint tissues necessitates precise control over the spatial arrangement and differentiation timing of multiple cell types. This complexity is particularly evident when co-culturing chondrocytes with osteoblasts or neurons, as each cell type has distinct maturation timelines and environmental requirements. Chondrocytes, the primary cells of cartilage, typically require approximately 21 days *in vitro* to progress through stages of proliferation, matrix production, and hypertrophy [139,140]. During this period, they express specific markers such as collagen type II and aggrecan, essential for maintaining cartilage integrity [141]. In contrast, osteoblasts, responsible for bone formation, undergo differentiation over a similar timeframe, with mineralization processes becoming prominent around days 14–21 days [142,143]. The overlapping maturation periods of these two cell types necessitate careful orchestration to prevent premature mineralization of cartilage regions, which can lead to pathological calcification. Besides, neurons that are derived from human pluripotent stem cells can take several weeks to months to achieve full maturation, characterized by extensive neurite outgrowth and synapse formation [144]. This extended maturation period contrasts with the relatively shorter differentiation timelines of chondrocytes and osteoblasts, posing challenges in synchronizing the development of all cell types within the same platform [145,146].

The joint is a highly compartmentalized multi-tissue system composed of distinct yet interacting microenvironments, including subchondral bone, articular cartilage, synovial membrane, and fibrocartilaginous structures. Each zone exhibits unique biochemical compositions, mechanical properties, oxygen tensions, and extracellular matrix (ECM) architectures, all of which are critical in maintaining tissue-specific functions. Replicating these discrete microenvironments within a single JoC platform is inherently challenging, particularly due to diffusion-driven homogenization of soluble factors and limited control over fluid dynamics at the microscale. For example, the subchondral bone niche favors an environment with stiff mechanical properties, to support mineralization and osteogenic activity. In contrast, the cartilage region requires softer mechanical properties to maintain chondrocyte phenotype and prevent hypertrophy. Failure to maintain this zonal distinction often results in undesired outcomes compromising the physiological relevance of the model. The situation becomes even more complex when incorporating neurons into JoC systems for studying joint innervation and pain pathways. Sensory neurons thrive under low mechanical loading conditions and require neurotrophic factor gradients (e.g., NGF, BDNF) for axonal guidance, which differ substantially from osteogenic and chondrogenic environments. Moreover, the mechanical stiffness and high ion concentration of osteogenic regions can inhibit neurite extension and lead to neurodegeneration if not properly isolated. Without effective localized control, diffusion of neurotoxic factors such as reactive oxygen species (ROS) or excessive calcium from osteogenic regions can adversely affect neuronal viability and function.

To overcome these limitations, advanced JoC systems are beginning to employ microfluidic barrier designs, hydrogel partitions with selective permeability, and precisely engineered flow patterns to establish and maintain spatially distinct biochemical and mechanical niches. Materials likeGelMA and hyaluronic acid-based hydrogels are often used to create ECM-like scaffolds that support tissue-specific environments while limiting undesired molecular crosstalk. Additionally, oxygen-impermeable barriers and on-chip oxygen control systems have been introduced to replicate the hypoxic conditions required by cartilage tissues while maintaining normoxic environments for bone regions. Despite these advances, achieving long-term stability of localized microenvironments remains a critical hurdle, especially under dynamic perfusion conditions required for nutrient exchange and waste removal.

### 6.2 Automatic operation mode

An automatic control mode is beneficial for ‘adaptive’ physiological simulation on a joint-on-a-chip. Human activities such as walking and running impose highly dynamic and variable compression forces with distinct frequencies and amplitudes on the joint. Simple, static compression protocols are insufficient to mimic this physiological complexity. Sophisticated, large-scale mechanical actuators are well-established in macroscale implant testing to precisely control load magnitude and frequency [147]. However, they are fundamentally incompatible with microfluidic-based JoC platforms due to their size and operational requirements. Current JoC systems often rely on simplistic compression regimes, which fail to capture this dynamism.

Automated manipulation is centered around solving the problem of automated fluid handling. Traditional microfluidics rely on bulk pumping systems to realize the liquid transport on a chip. In recent decades, scientists have extensively explored the natural mechanisms of spontaneous fluid transport in animals and plants and developed various directional fluid transport systems [148,149]. These methods support the programmability of liquid transport, which serves as the essence of automation. By adopting different external stimuli, many digital microfluidics have been reported to programmably manipulate discrete droplets, which includes moving, splitting, merging and mixing droplets on planar without any pumps and channels [[150], [151], [152]]. Although the application of digital microfluidics in biology is still in the exploratory stage, its pipeline-free delivery mode can greatly reduce the shear effect of solid walls on cells. For example, Wang et al. [153] reported capillary wrapping-assisted droplet manipulation with low movement resistance, which enables cell spheroid formation and high throughput drug screening. However, when applied to organs-on-chips, it is necessary to consider the design of nutrient solution delivery from a biological perspective to ensure long-term cell culture and interaction between tissues without crosstalk and reduce the impact of external stimuli on tissues. While processing improvements have streamlined photolithography-based manufacturing, the need to tightly clamp each individual casting remains an inevitable and persistent bottleneck. Current methods show various strategies to avoid fluid leakage during fluid infusion by chemical bonding, mechanical pressing, or adding leak-proof rubber tubes, which have been discussed in detail [154].

Artificial intelligence (AI), as a key tool for achieving intelligent fluid control, provides a viable technical path to this end. In recent years, AI has revolutionized data processing and analysis, and has been widely applied in microfluidic platforms, such as sensing enhancement, synthetic biology regulation, and non-invasive health monitoring using skin-interface microfluidic devices [155]. Although systematic reports on this technology have yet to be published in the organ-on-chip field, intelligent microfluidics has demonstrated potential for automated analysis of experimental processes, system optimization, and efficiency improvements. For example, a study by Bhuiyan et al. demonstrated automatic manipulation and targeted bubble elimination using AI-controlled microfluidic platforms [156]. In the specific application of joint-on-chips, the integration of AI has two important implications: first, it can assist in optimizing chip structural design and device operating parameters; second, it has the potential to construct automated control systems based on cell state-nutrient delivery feedback, enabling dynamic adjustment of culture conditions such as flow rate and shear force, thereby enhancing the biomimetic nature of the model and experimental reproducibility.

### 6.3 Sensors integration

Embedding biosensors into JoCs enables real-time monitoring of tissue behavior, inflammatory markers, or mechanical forces. These signals are vital for dynamic studies of disease progression and drug response. The ideal sensing system needs to meet the requirements of biocompatibility, mechanical flexibility, miniaturization and scalable manufacturing. Traditional sensor technologies, such as electrochemical, optical, and piezoelectric sensors, are typically for specific signal monitoring Therefore, integrating multiple sensors into one chip is undoubtedly a burden for microchips. To this end, a functional sensor unit based on hybrid mechanisms that can monitor multiple parameters facilitate chip integration [157]. To accelerate data analysis, raw physical/chemical signals such as current, voltage, or impedance generated by sensors can be used to construct complex nonlinear mappings between them and the physiological state of cells using artificial intelligence algorithms.

Another critical limitation is that many commercial sensors are rigid and planar, making them unsuitable for conformal integration within the three-dimensional architectures of JoC devices. For instance, embedding strain gauges or mechanical stress sensors to monitor real-time joint loading is technically challenging without disrupting fluid dynamics or compromising the soft tissue-like mechanical properties of the device. Similarly, integrating electrochemical sensors for inflammatory cytokines (e.g., IL-6, TNF-α) or cartilage degradation markers (e.g., MMP-13, COMP) require stable and specific functionalization of the sensor surface, which can degrade over time under continuous flow and biological conditions. This leads to sensor drift and reduced measurement accuracy over extended experimental periods. Emerging solutions include the development of flexible and printable sensor technologies based on conductive inks like liquid metal, graphene-based materials, and organic semiconductors, which allow for low-profile integration directly onto microfluidic substrates [158].

Furthermore, these sensors are typically integrated through manual assembly or post-fabrication modifications, which are not scalable for high-volume production. To address scalability concerns, researchers are also exploring modular sensor cartridges that can be easily inserted into JoC platforms, decoupling the sensor fabrication from the chip manufacturing process [159]. This approach simplifies replacement and reduces the risk of sensor failure affecting the entire experiment. However, the challenge remains in ensuring tight integration and accurate spatial localization of these sensors relative to the biological tissues of interest. Ultimately, widespread adoption of real-time biosensing in JoC will require innovations that combine biocompatibility, mechanical flexibility, miniaturization, and manufacturing scalability.

### 6.4 High-resolution imaging in JoCs

Real-time imaging is essential to evaluate cellular morphology, tissue remodeling, and matrix deposition in JoCs. High-resolution imaging is fundamental to evaluating cellular morphology, tissue remodeling, and extracellular matrix (ECM) deposition within Joint-on-Chip (JoC) platforms. Imaging also plays a critical role in assessing dynamic cellular behaviors such as migration, proliferation, and interactions between different tissue types. Despite its importance, the materials traditionally used for JoC fabrication pose significant limitations for advanced imaging modalities, including confocal, multiphoton, and super-resolution microscopy.

High resolution is essentially affected by the light transmittance properties of the material. PDMS exhibits good transparencies for many modes of optical imaging platform. However, with growing interest in scaling up PDMS manufacturing and minimizing manual assembly, multiple alternative materials have been explored. COC and PMMA demonstrate high optical clarity and lower autofluorescence. Moreover, the 3D complexity of JoC platforms adds additional imaging challenges. As tissue models become more physiologically relevant with thicker ECM deposition and multilayered cell structures, light scattering and absorption significantly reduce image quality, particularly for deeper tissue regions. Advanced imaging modalities such as multiphoton microscopy and light-sheet fluorescence microscopy can mitigate some of these issues but require JoC materials with high optical homogeneity and minimal scattering properties—criteria that many current microfabrication materials fail to meet.

To overcome these challenges, researchers are exploring the development of optically transparent, bioinert hydrogels and advanced polymers specifically engineered for low autofluorescence and high refractive index compatibility with biological tissues. Additionally, hybrid devices combining glass-bottom wells with polymeric microfluidic channels are increasingly adopted to balance imaging quality with design flexibility. Despite these advances, achieving universal, cost-effective material that meets both biological and optical requirements remains an open challenge for the widespread implementation of high-content imaging in JoC platforms.

### 6.5 A vision for next-generation JoC

When studying other factors affecting joint disorders, many JoCs overlooked the synergistic effects of compression and stretching associated with movement. Current joint chips often only support compression or stretching. Chips that fully simulate the physiological mechanical environment of joints remain a future goal. Furthermore, joints are composed of multiple tissues, requiring consideration not only of the interactions within compartmentalized compartments but also of the interactions between multiple tissues at their interfaces, such as the bone-cartilage interface. We envision the future development of a multifunctional, high-resolution tissue-engineered *in vitro* JoC model that integrates fluid perfusion, programmable compression control, and high-resolution tissue engineering. Ideally, this platform would support long-term cell culture, *in situ* cell status monitoring, and the integration of micro-biochemical sensors for long-term monitoring of biochemical and cellular signals.

## 7 Conclusion

The developmental trajectory of Joint-on-Chip (JoC) platforms is inherently multidisciplinary, with progress built upon advances across biomaterials, microfabrication, bioengineering, and sensor technologies. In this review, we summarized recent microfluidic fabrication methods to facilitate readers to choose the appropriate processing technology according to joint biology. Existing methods have made great progress in high-precision printing and expected biocompatibility. In the future, more refined processing technology innovations and new biomaterials are needed to reproduce *in vitro* more closely to biological tissues, such as capillaries at the scale of 10 μm, and long-term *in vitro* biomimetic tissue activity. In addition, representative cell types in various tissues are used as biomimetic units to study tissue interactions. It is feasible to complete physiological and pathological studies within the effective period of cells that do not differentiate at the laboratory level. Although this can effectively balance physiological complexity and experimental practicality, JoC models that are more representative of the *in vivo* environment still need to be highlighted in the future.

## CRediT authorship contribution statement

**Xuejiao Wang:** Writing – review & editing, Writing – original draft, Visualization, Investigation, Conceptualization. **Louis Jun Ye Ong:** Writing – original draft, Conceptualization. **Marcel Karperien:** Writing – review & editing. **Chunyi Wen:** Writing – review & editing, Conceptualization.

## Declaration of competing interest

The authors declare no conflict of interest in this work.

## Data Availability

No data was used for the research described in the article.
